# DLM-DTI: a dual language model for the prediction of drug-target interaction with hint-based learning

**DOI:** 10.1186/s13321-024-00808-1

**Published:** 2024-02-01

**Authors:** Jonghyun Lee, Dae Won Jun, Ildae Song, Yun Kim

**Affiliations:** 1 Department of Medical and Digital Engineering, Hanyang University College of Engineering, 222, Wangsimni-ro, Seongdong-gu, Seoul, 04763 Korea; 2 Department of Internal Medicine, Hanyang University College of Medicine, 222, Wangsimni-ro, Seongdong-gu, Seoul, 04763 Korea; 3 Department of Pharmaceutical Science and Technology, Kyungsung University, 309, Suyeong-ro, Nam-gu, Busan, 48434 Korea; 4 College of Pharmacy, Deagu Catholic University, 13-13, Hayang-ro, Hayang-eup, Gyeongsan-si, 38430 Gyeongsangbuk-do Korea

**Keywords:** Drug-target interactions, Pre-trained language model, Knowledge adaptation, Lightweight framework

## Abstract

The drug discovery process is demanding and time-consuming, and machine learning-based research is increasingly proposed to enhance efficiency. A significant challenge in this field is predicting whether a drug molecule’s structure will interact with a target protein. A recent study attempted to address this challenge by utilizing an encoder that leverages prior knowledge of molecular and protein structures, resulting in notable improvements in the prediction performance of the drug-target interactions task. Nonetheless, the target encoders employed in previous studies exhibit computational complexity that increases quadratically with the input length, thereby limiting their practical utility. To overcome this challenge, we adopt a hint-based learning strategy to develop a compact and efficient target encoder. With the adaptation parameter, our model can blend general knowledge and target-oriented knowledge to build features of the protein sequences. This approach yielded considerable performance enhancements and improved learning efficiency on three benchmark datasets: BIOSNAP, DAVIS, and Binding DB. Furthermore, our methodology boasts the merit of necessitating only a minimal Video RAM (VRAM) allocation, specifically 7.7GB, during the training phase (16.24% of the previous state-of-the-art model). This ensures the feasibility of training and inference even with constrained computational resources.

## Introduction

The process of drug discovery is often compared to finding a needle in a haystack, requiring substantial funds and labor forces. Unfortunately, most newly discovered drugs fail to obtain approval for clinical use due to unexpected adverse drug reactions, insufficient drug effects, and low binding affinity [1–5]. Artificial intelligence has emerged as a promising tool for reducing expenses in various fields of drug discovery, including the predictions of drug toxicity, drug-drug interaction, and molecule properties, among others. In the first step of drug discovery, which involves drug repurposing and/or repositioning, it is critical to identify candidates of druggable molecules that target a specific protein. In this context, drug-target interaction (DTI) prediction tasks have emerged as a crucial area of research.

Previous studies on DTI prediction can be broadly categorized into three categories: simulation-based molecular docking, structural similarity, and deep neural network (DNN) approach. Molecular docking simulation utilized 3D structures of proteins and molecules and simulated the binding sites [6–8]. While it offers a clear visual understanding, obtaining a 3D structure of a feature is challenging and it was hard to collect large datasets effectively. Conversely, the similarity-based technique proposed binding candidates using priorly established drug-target pairs. While this approach showed considerable predictions for recognized pairs based on similarity, it confronts difficulties in determining similarity for previously unobserved pairs [9, 10]. DNNs have exhibited proficient results in DTI prediction, similar to their successful implementations in various other domains. A pioneering study, DeepDTA [11], employed a drug and target encoder built on Convolutional Neural Networks (CNN) for the prediction of binding affinities. Instead of relying on highly complex datasets, the DeepDTA leveraged 1D expressions of the molecular structure system, Simplified Molecular Input Line Entry System (SMILES), and amino acid sequences, for drug and target, respectively. With hierarchical CNN layers, similar to conventional CNNs used for image recognition, DeepDTA can interpret the interactions of a given drug-target pair. After the DeepDTA, a multitude of research initiatives have been undertaken to either enhance the encoder’s capability or predict interactions more effectively. Such advancements encompass the deployment of CNNs [12–14], the development of interactions within gated cross attentions [15], the adoption of encoders that perceive molecular structures in graph format [16–18], computing similarity using enhanced DNN-based kernels [19–21], encode sequence using generative models [22, 23], and the integration of multi-modal techniques [24–27].

The Transformer architecture [28], renowned for its proficiency in sequence processing, has been extensively employed as an encoder [29–37]. Nonetheless, it possesses a fundamental limitation: the computational expense escalates quadratically with the increase in the input length (see more details in Appendix C). Consequently, a majority of research initiatives have leaned towards its application as a drug encoder rather than for proteins [30–33, 37]. Recent advancements have brought forth efficient transformer methodologies, suggesting the potential for significantly reducing the computational demands in protein-encoding [38–41]. Concurrently, the ProtTrans project [35], leveraging the established Bidirectional Encoder Representations from Transformers (BERT) [42] model and its training methodology has undertaken pre-training of a protein encoder using an expansive set of amino acids and subsequently made it publicly available. As of now, the academic community lacks a publicly accessible, pre-trained model based on the efficient transformer, thereby preserving the relevance and utility of ProtTrans. A recent study, that utilized both transformer-based encoders for representing drugs and targets was proposed [43]. The prediction performances were considerably improved, however, due to the large size of the protein encoder, they truncated the protein language model into half its size.

To reach an efficient computing model, knowledge distillation techniques were proposed [44, 45]. The key concept of knowledge distillation is distilling the knowledge from the large and complex model to the small and simple model with minimum loss of knowledge (See more details on Appendix A). However, DistillProtBERT (260 million parameters) [46], a model employing knowledge distillation from ProtBERT (420 million parameters) [35], is less efficient due to the inherent complexity of the amino acid sequence.

To address this, we proposed a more efficient learning method than knowledge distillation, namely hint-based knowledge adaptation. This method involves using the intermediate features of the teacher model as hints, representing an expansion of knowledge distillation inspired by FitNet [47]. We term this approach “general knowledge” as it provides a general understanding of the target sequence, though lacking direct knowledge of the DTI task. It is assumed that this general knowledge, serving as a hint to the sequence, will facilitate successful learning despite the small size and simplicity of the student model. Conversely, the student model, designed to directly learn DTI performance, was structured in a simplified form compared to the original ProtBERT. In essence, knowledge adaptation presents an efficient means of leveraging both general knowledge of the target sequence and task-specific knowledge related to DTI simultaneously. This underscores the concept of adapting the teacher’s knowledge to the student’s knowledge, in contrast to knowledge distillation, which directly conveys task-specific knowledge.

In this study, we proposed a Dual Language Model-based DTI model named DLM-DTI. The DLM-DTI was a lightweight and efficient, but accurate DTI prediction model. With the knowledge adaptation, the rich information from ProtBERT successfully adapted to predict DTI tasks. This study has several key contributions:

1. The hint-based knowledge adaptation technique, despite its compact parameterization, demonstrates considerably improved performance compared to baseline methods.
2. By utilizing cached outputs from the teacher network, we achieved a notable reduction in computational costs.
3. The knowledge adaptation approach is model-agnostic, offering flexibility in the selection of pre-trained models and architectures.

## Materials and methods

### Problem definition

In binary DTI classification, the goal is to predict the target value, $Y_i$, for a given pair of $X_i$, where $\text {X}_i = \{ \text {x}_{\textrm{drug}}^i, \text {x}_{\textrm{target}}^i \}$, and $\text {Y}_i \in \{ 0, 1 \}$ for $i=1,\cdots , N$. The prediction of DTI can be viewed as a mapping function $f(X_i) \rightarrow [0,1]$, which maps the drug-target pairs to a probability score of the interaction.

### Sequence representation

**Fig. 1:**
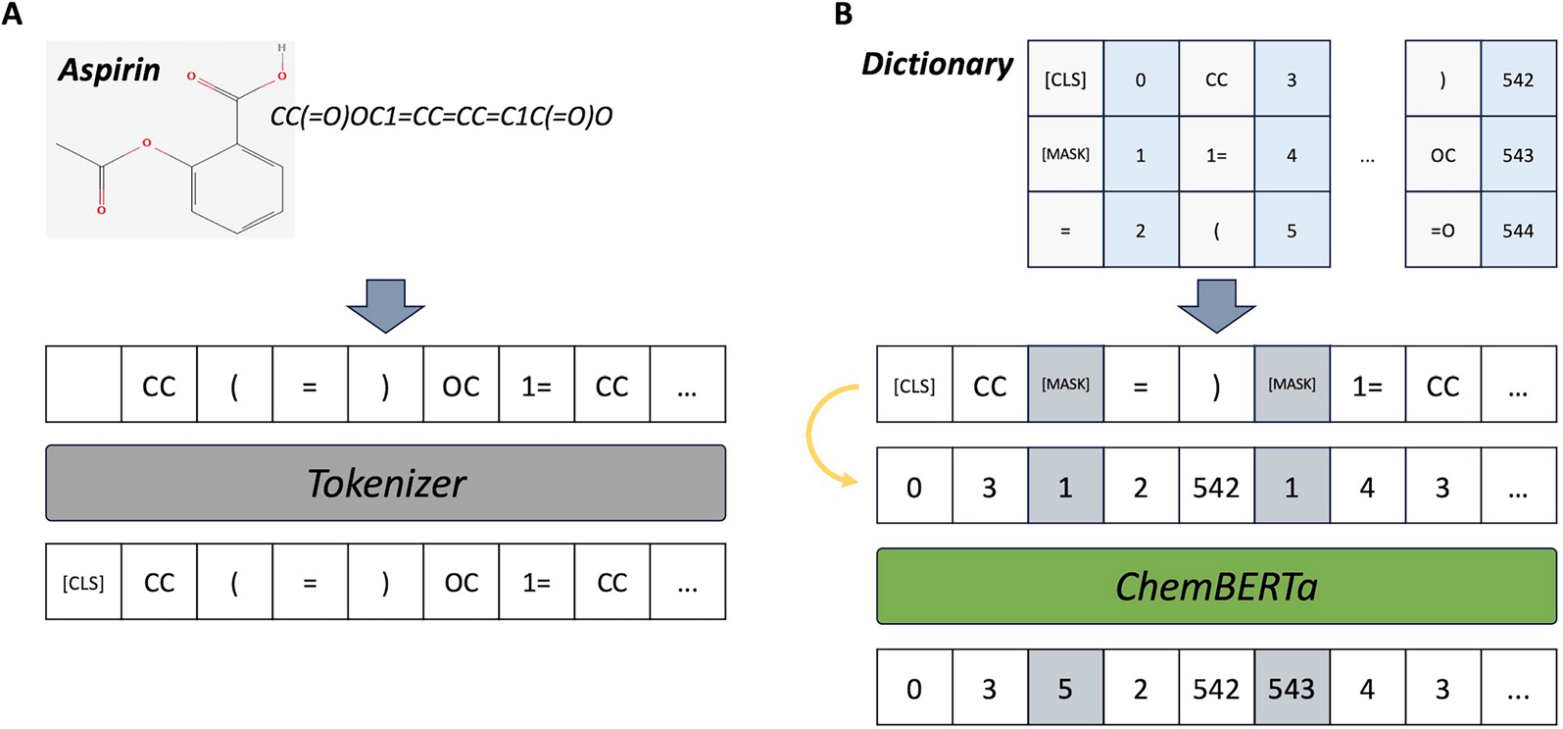
The concept of sequence representation and pre-training is illustrated. In **A**, the tokenization of a drug sequence (SMILES string) is depicted. In **B**, the tokenized elements are converted into integer values according to the predefined dictionary, and the encoder model (in this example, ChemBERTa) restores masked tokens into the original tokens (tokens colored in gray). After pre-training, the class token (CLS) is used to represent a given sequence

Sequence representations and embeddings involve converting a sequence, like a sentence, into a format that a computer model can understand. The first step is turning each part of the sequence into tokens, which are basically integer numbers that the model can work with. In this study, each part of the sequence is treated as a separate token. Special tokens, like a class token, are also added to grasp the overall meaning of the entire sequence. The concept of tokenization and special tokens is illustrated in Fig. 1.

### Dataset configurations

We employ three datasets, namely DAVIS, Binding DB, and BIOSNAP, to train and evaluate the DLM-DTI. The DAVIS dataset consists of 68 drugs and 379 proteins, with 11,103 interactions measured in dissociation constant (${K}_{d}$) [48]. The interactions are categorized as positive or negative, with 1506 and 9597 instances, respectively. Similarly, the Binding DB dataset includes 10,665 drugs and 1413 proteins, with 32,601 interactions measured in ${K}_{d}$ [49]. The interactions are categorized as positive or negative, with 9166 and 23,435 instances, respectively. In this study, the threshold value for ${K}_{d}$ is set to 30 units, and interactions with ${K}_{d}$ values less than 30 units are considered positive binding interactions between the given drug and protein pair [29, 43]. The BIOSNAP dataset is initially composed of positive interactions only; however, negative pairs are added in the MolTrans study. The BIOSNAP dataset comprises 4510 drugs and 2181 proteins, with 27,482 interactions, including 13,741 positive and 13,741 negative instances [29].

The integrated data training was first proposed by Kang et al., and they demonstrated improvements [43]. In this setting, training and validation datasets were merged, and a model was trained using integrated datasets. After the training steps, the trained model with integrated training datasets was evaluated on individual test datasets. For example, to test the BIOSNAP test dataset, the model was first trained using DAVIS, Binding DB, and BIOSNAP’s training datasets, and then tested on BIOSNAP’s test dataset. Generally, the diversity and quantity of datasets are linked to the improvement of prediction performance. Therefore, we also assessed the impact of dataset integrations using DLM-DTI. A summary of the dataset description is presented in Table 1.

**Table 1:** The description of datasets

Dataset	Drugs	Targets	Interactions	
			Positive	Negative
DAVIS	68	379	1506	9597
Binding DB	10,665	1413	9166	23,435
BIOSNAP	4510	2181	13,741	13,741
Integrated^1^	11,700	3067	24,413	46,773

^1^ The unique values after integaration

To ensure a fair comparison of model performance, we employ the same training, validation, and testing datasets used in previous studies [29, 43]. The datasets are split into training, validation, and testing datasets in the ratio of 7:1:2, respectively. The number of interactions for each data splitting is summarized in Table 2.

**Table 2:** The number of interactions for each split

Setting	Training	Validation	Testing
DAVIS	2086	3006	6011
Binding DB	12,668	6644	13,289
BIOSNAP	19,238	2748	5496
Integrated^1^	33,992	12,398	(6011/13,289/5496)

^1^ Training and validation are conducted using merged datasets; however, testing is performed on individual datasets

### Model configurations

**Fig. 2:**
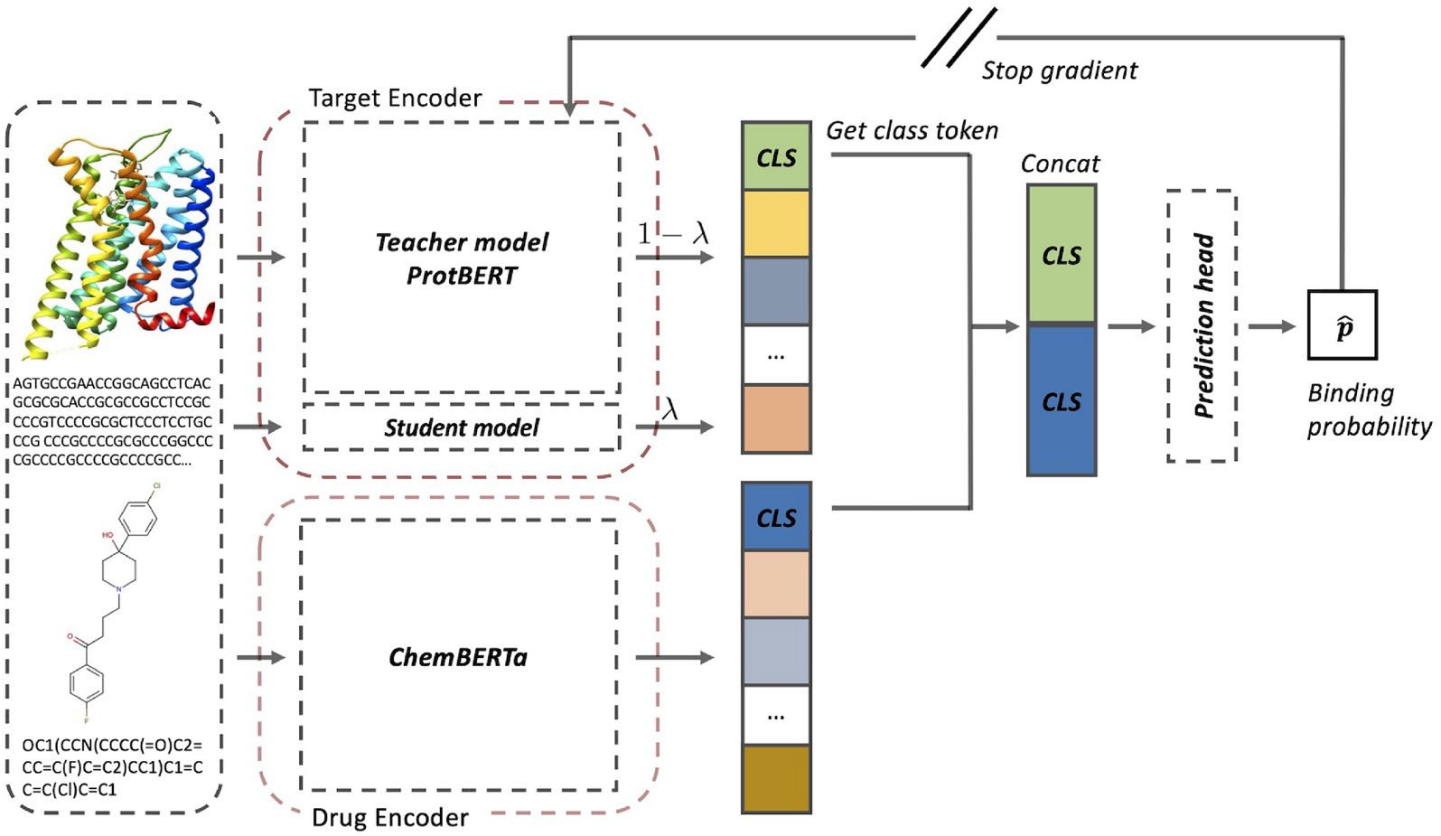
The process flow of DLM-DTI. The drug and target sequences feed into their respective encoders. The encoded sequences are then merged, and the probability of bindings is computed using the interaction prediction head. DLM-DTI only utilizes the class token (CLS) of each encoded sequence because the class token preserves the abstract meaning of the entire sequence. The features of target sequences are computed using a teacher-student-based architecture, specifically employing a hint-based learning strategy

The process flow of DLM-DTI is depicted in Fig. 2. DLM-DTI was comprised of three primary components: the drug encoder, target encoder, and interaction prediction head. Notably, the target encoder encompasses both the teacher and student models of language models for protein sequences.

#### Drug encoder

The drug encoder converts SMILES sequences into meaningful features, serving as a mapping function from molecule sequences to a meaningful chemical space. We employed the ChemBERTa encoder, which was trained on various canonical SMILES and learned chemical space. Further details are described in Appendix B.

The class token of the last hidden layer was extracted as input for the interaction prediction head. The encoding process of the drug sequence can be represented as follows:

1 $$\begin{aligned} z_{\textrm{drug}} = f\left( \text {LN}(x_{\textrm{class}})\right) , \end{aligned}$$

where $\text {LN}(\cdot )$ denotes the layer normalization layer, $f(\cdot )$ denotes the projection function used to align the dimensions, and the hidden dimensions were set to 512 in this study. The upper limit of the drug sequence length was 512 tokens, corresponding to the maximum sequence length of the original ChemBERTa encoder [31].

#### Target encoder

Similar to the drug encoder, the target encoder also extracts meaningful features from raw target sequences (amino acid sequences). The target encoder in this study was composed of both a teacher and a student model. The teacher model used for target sequence encoding was the ProtBERT model, pre-trained on UniRef and big fantastic database databases [35]. Details of ProtBERT are described in Appendix C. The original ProtBERT model was trained on sequences up to 40 K characters, with 420 million parameters. The student model was designed to match the original teacher model, ProtBERT, however, the number of layers was reduced. Except for the number of layers, the student model followed the hyperparameters of the teacher model. The number of parameters of the student model was 6.2% of the teacher model; teacher model: 420.0 million, student model: 26 million. The detailed parameters of the target encoder are presented in Table 3.

**Table 3:** The specific parameters of target encoder

	Teacher	Student
Number of hidden layers	30	2
Number of attention heads	16	16
Hidden dimension	1024	1024
Intermediate-size	4096	4096
Number of parameters	420 M	26 M

In most cases, fully fine-tuning the large model was impractical due to restrictions on datasets and the associated computational expenses. To address this challenge, we adopted a hint-based training scheme that kind of knowledge distillation comprises both a teacher model and a student model. The teacher model was prevented from parameter updates, enabling solely the parameters of the student model to be updated. Given that the teacher model’s output was not subject to training, it retained a fixed form, thus enabling us to cache outputs of the teacher model prior to the training and inference step. This strategy markedly minimizes computational redundancy, thereby optimizing computational efficiency. Considering the teacher model’s output was not trained, it served as a form of hint to which the task-specific model (student model) could refer. The teacher and student models were combined using class token mixing to encode the target sequence. The output class token was treated as a “hint” that contained general knowledge of the given protein sequence. On the other hand, the output class token of the student model was considered as task-oriented specific knowledge. To mix the general knowledge and task-specific knowledge, we added two class tokens with learnable gating parameters ($\lambda$). The encoding process of the target sequence can be represented as follows:

2 $$\begin{aligned} z_{\textrm{target}} = \lambda g\left( \text {LN} (x^{\textrm{student}}_{\textrm{class}})) + (1 - \lambda ) h (\text {LN}(x^{\textrm{teacher}}_{\textrm{class}})\right) , \end{aligned}$$

where $g(\cdot )$ and $h(\cdot )$ are the projection functions used to align the dimensions, and the adaptation parameter $\lambda$ is a learnable parameter initialized randomly from a uniform distribution, $\lambda \sim Uniform(0, 1)$. The term “adaptation” was employed to describe the process of adjusting general knowledge to suit the specific requirements of a particular task. An elevated value of the adaptation parameter indicated an increased emphasis of the model on the class token derived from the teacher model. In contrast, a decreased value of the adaptation parameter signified a predominant utilization of task-specific information obtained from the student model. The hidden dimensions of the class token mixing were set to 1024 in this study. The maximum length of the target sequence was set to 545 tokens, which covered 95% of proteins in the datasets, and the same max protein sequence lengths of previous studies [29, 43].

#### Interaction prediction head

The class tokens of drug and target sequences have abstract meanings for each sequence. The interaction prediction head aggregated the features of drug-target pairs and predicted binding probability. In this step, there were multiple choices for mixing the features; for example, cross attention, capsule network, etc. However, we simply employed concatenation that showed stable performances in the previous work [43].

**Fig. 3:**
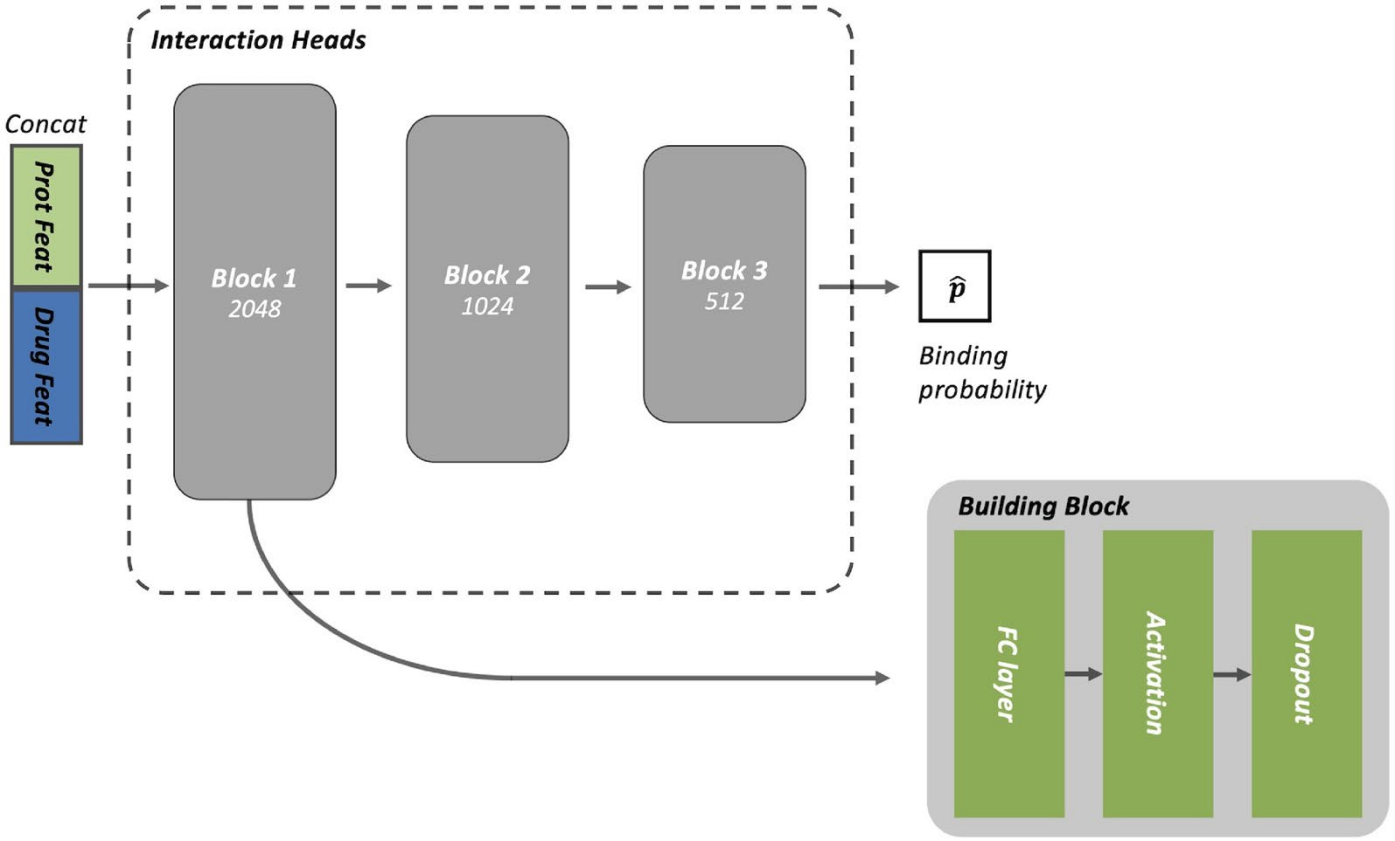
Structure of the interaction prediction head. The interaction prediction head mixes the features of the drug-target pair to predict the binding probability of a given pair. The number under the block indicates the feature dimension

The interaction module consists of three sequential blocks. Each block is structured with a Fully Connected (FC) layer, followed by an activation function and subsequently a dropout layer. The respective dimensions of the FC layers are 2048, 1024, and 512. The chosen activation function for these blocks is the Gaussian Error Linear Unit (GeLU). Additionally, a dropout rate of 0.1 has been employed for regularization. A detailed schematic of this configuration can be found in Fig. 3, and the specific parameter values are summarized in Table 4.

**Table 4:** The detailed parameters of interaction prediction head

Block	Layers	Input dimensions	Output dimensions	Dropout rate
Input	Concatenation	512^1^, 512^2^	1024	
Block 1	FC layer	1024	2048	
	GeLU & Dropout	2048	2048	0.1
Block 2	FC layer	2048	1024	
	GeLU & Dropout	1024	1024	0.1
Block 3	FC layer	1024	512	
	GeLU & Dropout	512	512	0.1
Output	FC layer	512	1	

^1^ Feature of drug sequence, $z_{\textrm{drug}}$

^2^ Feature of target sequence, $z_{\textrm{target}}$

### Experimental setup

#### Evaluation metrics

We used the Area Under the Receiver Operation Characteristics curve (AUROC) and the Area Under the Precision-Recall Curve (AUPRC) as primary evaluation metrics. AUROC is one of the most favorable metrics to measure classification performance, particularly in the medical field; however, it could be easily overestimated when the data has class imbalance [50]. Therefore, AUPRC is a relatively robust metric for measuring classification performance in imbalanced settings [50]. Sensitivity and specificity scores were utilized as sub-metrics, and the threshold for these sub-metrics was simply set to 0.5.

#### Model training hyperparameters

The DLM-DTI was optimized using the AdamW optimizer with a learning rate of 0.0001. A cosine annealing learning rate scheduler was employed to adjust the learning rate. The binary cross-entropy loss was used to calculate the difference between predictions and ground truth. The model was trained for 50 epochs, and the best-performing parameters were selected based on the AUPRC score during validation. Due to severe class imbalance, the model could easily be overfitted to the dominant class. To prevent the selection of an overfitted model, we set the selection criteria as AUPRC rather than AUROC or the minimum loss coefficient. Automated mixed precision was utilized, and the batch size was set to 32. The best combination of hyperparameters was determined through iterative experiments.

The use of a class imbalance sampler did not show any benefit for model training; therefore, we did not apply an imbalance sampler. Instead, AUPRC-based optimization demonstrated better performance in predicting binding probability.

#### Hardware and softward

We used a single NVIDIA A100 GPU to train DLM-DTI. The Python (v3.8) and PyTorch deep learning framework (v1.13) for trained DLM-DTI.

## Results

### Binding probability prediction

The baseline models, namely MolTrans [29] and the approach by Kang et al. [43], along with our proposed DLM-DTI, were trained on the same training datasets and evaluated using identical test datasets. Table 5 presents a summary of the evaluation results obtained from these experiments. MolTrans was exclusively trained on individual datasets and evaluated individually. In contrast, both Kang et al. and our DLM-DTI were trained using both individual and combined dataset settings. This approach was claimed in Kang et al., and therefore the previous study, MolTrans, did not experiment with an integrated dataset.

Within the BIOSNAP dataset, DLM-DTI showed an improved AUPRC score (absolute value; percentage) than MolTrans (0.013; 1.44%), and Kang et al. (0.014 $\sim$ 0.017; 1.56 $\sim$ 1.90%). The AUROC score was improved compared to MolTrans (0.019; 2.12%), however, the AUROC showed similarity to Kang et al.’s model. Similarly, in the Binding DB, DLM-DTI exhibited a considerably improved AUPRC score than other methods, MolTrans (0.021; 3.38%), and Kang et al.’s model (0.004 $\sim$ 0.02; 0.63 $\sim$ 3.21%), respectively.

In the DAVIS dataset, the performance of the DLM-DTI was degraded, and its performance was similar to that of MolTrans. The training with an integrated dataset showed benefits for the DLM-DTI only in the DAVIS dataset.

**Table 5:** The prediction performance of binding affinity

Dataset	Model	AUROC	AUPRC	Sensitivity	Specificity
BIOSNAP	MolTrans	0.895 ± 0.002	0.901 ± 0.004	0.775 ± 0.032	0.851 ± 0.014
	Kang et al., S	0.914 ± 0.006	0.900 ± 0.007	**0.862** ± **0.025**	0.847 ± 0.007
	Kang et al., I	0.910 ± 0.012	0.897 ± 0.014	0.830 ± 0.029	**0.863** ± **0.011**
	DLM-DTI, S	**0.914** ± **0.003**	**0.914** ± **0.006**	0.848 ± 0.016	0.844 ± 0.024
	DLM-DTI, I	0.910 ± 0.005	0.914 ± 0.004	0.850 ± 0.014	0.821 ± 0.006
DAVIS	MolTrans	0.907 ± 0.002	0.404 ± 0.016	0.800 ± 0.022	0.876 ± 0.013
	Kang et al., S	0.920 ± 0.002	0.395 ± 0.007	0.824 ± 0.026	**0.889** ± **0.015**
	Kang et al., I	**0.942** ± **0.005**	**0.517** ± **0.017**	**0.903** ± **0.017**	0.866 ± 0.015
	DLM-DTI, S	0.895 ± 0.003	0.373 ± 0.017	0.833 ± 0.044	0.802 ± 0.070
	DLM-DTI, I	0.898 ± 0.026	0.406 ± 0.026	0.860 ± 0.016	0.786 ± 0.022
BindingDB	MolTrans	0.914 ± 0.001	0.622 ± 0.007	0.797 ± 0.005	0.896 ± 0.007
	Kang et al., S	0.922 ± 0.001	0.623 ± 0.010	0.814 ± 0.025	0.916 ± 0.016
	Kang et al., I	**0.926** ± **0.001**	0.639 ± 0.018	0.802 ± 0.022	**0.928** ± **0.013**
	DLM-DTI, S	0.912 ± 0.004	**0.643** ± **0.006**	**0.888** ± **0.014**	0.793 ± 0.015
	DLM-DTI, I	0.912 ± 0.004	0.636 ± 0.007	0.869 ± 0.023	0.811 ± 0.010

S: single dataset, I: integrated dataset

Performances of five randomly initialized runs were averaged

Best performance is highlighted in bold

### Adaptation parameter, $\lambda$

**Fig. 4:**
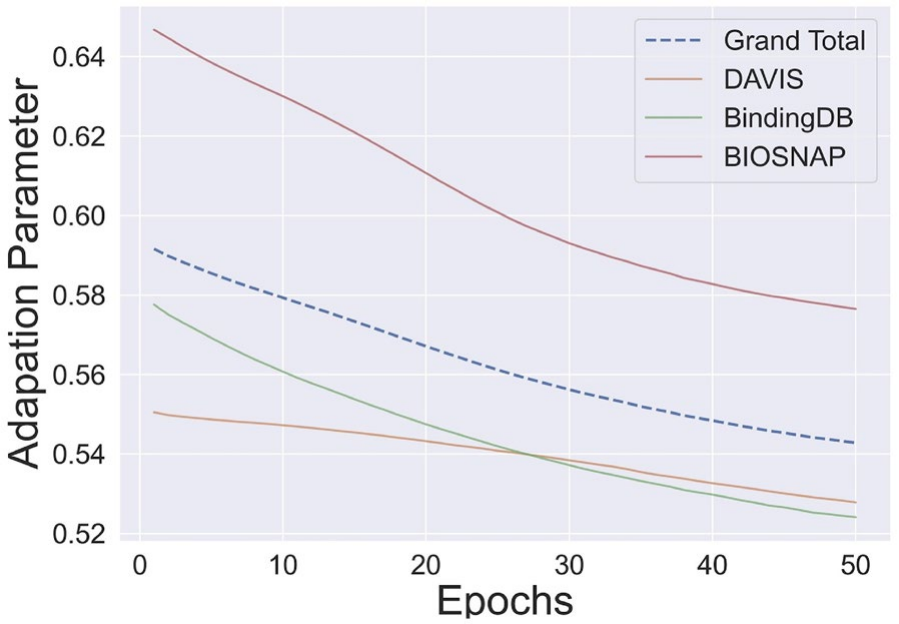
Variation of the adaptation parameter ($\lambda$) during model training process

During the training, the randomly initialized adaptation parameter $\lambda$ gradually decreased and converged, as illustrated in Fig. 4. The adaptation parameter controlled the feature weights from the teacher and student encoder. As mentioned earlier, the teacher encoder contained general knowledge of the target sequence, and the student encoder had narrow but specific task-related knowledge. With the adaptation parameter, the DLM-DTI modulated the importance of each feature to accurately predict binding probability.

To evaluate the effect of teacher-student architecture-based target sequence encoding, two ablation studies were conducted.

- $\lambda$ set to 0: Only the teacher encoder (general knowledge) was utilized.
- $\lambda$ set to 1: Only the student encoder (task-specific knowledge) was utilized.

The adaptation setting (which utilized both teacher-student encoders) showed the best performance (AUROC: 0.912; AUPRC: 0.643) compared to the teacher encoder-only setting (AUROC: 0.911; AUPRC: 0.635) or the student encoder-only setting (AUROC: 0.900; AUPRC: 0.635). The effect of the $\lambda$ parameter is summarized in Table 6.

**Table 6:** The prediction performance of binding affinity

	AUROC	AUPRC	Sensitivity	Specificity
Student only	0.900 ± 0.001	0.612 ± 0.003	0.845 ± 0.024	**0.805** ± **0.018**
Teacher only	0.911 ± 0.002	0.635 ± 0.002	0.880 ± 0.012	0.800 ± 0.014
Adaptation	**0.912** ± **0.004**	**0.643** ± **0.006**	**0.888** ± **0.014**	0.793 ± 0.015

Performances of five randomly initialized runs were averaged

Best performance is highlighted in bold

BindingDB dataset is utilized

The student encoder-only setting exhibited the poorest prediction performance (Rank: $\text {3}^{\textrm{rd}}$). This implies that two layers of simple and shallow networks were not sufficient to capture the complex patterns and features of target sequences to accurately predict DTIs. However, the teacher encoder-only setting demonstrated comparable performance (Rank: $\text {2}^{\textrm{nd}}$). This suggests that the general knowledge of the teacher model has the potential to predict binding probability. The teacher encoder-only setting corresponds to linear probing, where the training strategy only updates the prediction head without adjusting the weights of the encoder [51, 52]. The prediction performance of linear probing is considered as an encoder’s existing knowledge.

### Time and memory analysis

Typically, a model’s performance exhibits a direct correlation with its parameter count, suggesting that larger models often yield superior outcomes. Nonetheless, this advantage comes with a caveat; substantial models necessitate considerable computational resources during both the training and inference stages. In light of this, we embarked on a systematic analysis comparing training time and parameter counts (Table 7). The metric for training time was derived by computing the mean learning time across three epochs, utilizing the Binding DB dataset.

**Table 7:** Time and memory analysis of baseline models and DLM-DTI

	Parameters (M)	Training time (S)^1^	Memory capacity (GB)^2^	AUPRC^3^
MolTrans	62.8	75.33 ± 3.51	5.9	0.622 ± 0.007
Kang et al.	353.0	631.00 ± 17.06	47.4	0.623 ± 0.010
DLM-DTI	86.7	63.00 ± 1.00	7.7	**0.643** ± **0.006**

M: millions, S: seconds, GB: Giga bytes

^1^ Mean ± SD

^2^ Batch size is matched to 32

^3^ Results of Binding DB with single training setting. Best performance is highlighted in bold

DLM-DTI showed the best AUPRC score (0.643), only with 24.56% (86.7 million) of parameters compared to the Kang et al. (353.0 million) [43]. Additionally, DLM-DTI required 7.7 GB video random access memory (VRAM), and 63.00 s for a single training epoch. It was 16.24% (47.4 GB), and 9.98% (631.00 s) of the Kang et al. [43]. The MolTrans required the smallest VRAM (5.9 GB), however, the AUPRC score (0.622) was slightly lower than DLM-DTI (0.643). In our experimental setting, DLM-DTI required 7.7 GB of VRAM, therefore, it could be trained on conventional graphic processing units (GPUs), not for high-performing research machines (See details on 2.5.2).

### Cold drug, target, and bindings

In addressing DTI challenges, the cold splitting testing approach is widely adopted [36, 53], primarily due to the inherent difficulties in dataset procurement and the paramount importance of achieving generalization for novel pairs. The term “cold splitting” pertains to scenarios where previously unseen drug-target interactions are involved, ones that were excluded from both the training and validation datasets. To simulate this condition, we conducted experiments where we isolated cold drugs, cold targets, and cold binding interactions from the test set of models trained to utilize the Binding DB dataset. We identified a total of 2,127 cold drugs and 136 cold targets. Specifically, a cold drug configuration encompasses all interactions associated with a cold drug, while a cold target configuration comprises all interactions associated with a cold target. The cold bindings were the interactions between cold drugs and cold targets, and only 114 pairs were identified. The performances of cold-splitting datasets are summarized in Table 8. DLM-DTI’s performance was comparable to the baseline models in the context of the cold drug, yet exhibited a minor deterioration to the cold target and was found to be most deficient in addressing cold binding. Conversely, Kang et al. [43] manifested commendable prediction capabilities across all testing scenarios. MolTrans [29] exhibited a performance metric closely mirroring Kang et al. in terms of AUROC, but fell short when evaluated using AUPRC.

**Table 8:** The classification performances within the cold splitting settings

	MolTrans		Kang et al.		DLM-DTI	
	AUROC	AUPRC	AUROC	AUPRC	AUROC	AUPRC
Cold Drug	0.853	0.562	**0.884**	**0.617**	0.850	0.584
Cold Target	0.841	0.668	**0.855**	**0.716**	0.789	0.527
Cold Binding	0.718	0.370	**0.744**	**0.448**	0.622	0.261

Best performance is highlighted in bold

## Discussion

In this study, we suggested a lightweight but accurate DTI prediction model, namely DLM-DTI. The main hurdle for utilizing protein sequence-based language models, such as ProtBERT [35], was heavy computing resource requirements. To comprehend the complex and long sequence of a protein, it needed heavy and large architectures and an intensive pre-training process. The DLM-DTI mitigated the computational burden caused by the protein encoder, by using a knowledge adaptation. DLM-DTI achieved improved AUPRC performance, especially in Binding DB (0.63 $\sim$ 3.38%), and BIOSNAP (1.44 $\sim$ 1.9%) datasets. The most interesting point was that DLM-DTI utilized only 25% of parameters (86.7 million) compared to the previous state-of-the-art model, Kang et al. (353 million) [43]. Additionally, DLM-DTI required only 7.7 GB of VRAM, and 63 s for each training epoch, that of 16.24%, and 9.98% of Kang et al. [43].

The Transformer-based language model has exhibited impressive capabilities across various applications, including molecular and protein sequences. However, pre-training has emerged as a key approach to further optimize the model’s functional and semantic relationship learning from large sequence datasets [35–37, 42, 43]. Despite the promising results, the computational cost of the language model increases significantly with the input length. To address this challenge, Kang et al. proposed a Kang et al. approach, which employed only half of the pre-trained target encoder [43]. The methodology employed by the ELECTRA-DTA model aligns closely with our approach [36]. In the ELECTRA-DTA framework, the features originating from the pre-trained drug encoder and protein encoder are individually averaged. Subsequently, these averaged features are compactly represented as a compressed feature vector. This vector is subsequently incorporated into a squeeze-and-excitation network, aiming to enhance the predictive capabilities of the model. Their approach can also be perceived as a tactical maneuver to circumvent the necessity of fine-tuning the complete encoder. However, it is important to note that we could not directly compare the prediction performance of our DLM-DTI approach to that of ELECTRA-DTA due to differences in the target tasks, with DLM-DTI using binary classification and ELECTRA-DTA using $pK_{d}$ regression.

In our study, we introduced an adaptation parameter to efficiently generate meaningful protein features. The adaptation parameter, denoted as $\lambda$, was randomly initialized and tuned. This parameter controlled the weights of knowledge from both the teacher model (providing general knowledge) and the student model (capturing task-specific knowledge). In the ablation studies (Table 6), the absence of knowledge adaptation resulted in significant degradation of performance for both the teacher-only and student-only settings. However, the DLM-DTI with knowledge adaptation exhibited weaknesses in generalization performance. Kang et al.’s [43] work also demonstrated strong performance under cold-splitting conditions (Table 8). In contrast, our DLM-DTI, which either matched or outperformed Kang et al. on the complete dataset, showed reduced effectiveness in cold-splitting evaluations, particularly concerning cold-binding interactions. This may be attributed to the over-reduction of the student model, limiting generalization performance. Inspired by recent examples that incorporate natural language-based prior knowledge to enhance prediction performance, we aim to improve our approach by adding natural language information related to the function of proteins in future work [54]. Interestingly, integrated dataset training did not prove beneficial for DLM-DTI. In Kang et al. [43], training with integrated datasets demonstrated outstanding performances. Large-scale Transformer-based architectures typically require a substantial amount of data to realize their full potential. However, DLM-DTI introduces a small-scale student model, and it is speculated that the small size was sufficient for effective learning.

Recently, foundation models based on large language models have been widely studied [55, 56]. A shared challenge between these models and protein sequence encoders pertains to the intricacies involved in fine-tuning. Due to the scarcity of annotated data and the extensive parameters within these models, innovative strategies for effective fine-tuning have been proposed. For instance, a method called low-rank adaptation (LoRA) [57], similar to our own approach, adopt a technique where only the adaptation layer is adjusted. This is achieved by integrating a low-rank adaptation layer, which eliminates the need for comprehensive fine-tuning across all layers. This approach proves to be more cost-effective and quicker to converge compared to the resource-intensive process of complete fine-tuning. Therefore, in our future study, we plan to compare the performances of a fine-tuning model using LoRA’s adaptation approaches. Furthermore, there is a need for enhancement in the design of the interaction head. Currently, this component is composed of a sequence of straightforward FC layers, which exhibits reduced effectiveness in cold bindings. To address this, potential strategies include the integration of a squeeze-and-excitation network [58], capsule network [59], cross-attention [60], and other alternatives.

## Conclusion

In this study, we employed knowledge adaptation to efficiently and accurately predict binding probability. The knowledge adaptation was efficiently tuned with both general knowledge and task-specific knowledge through the teacher-student architectures. With only 25% of the model parameters, DLM-DTI exhibited considerable performance compared to the previous state-of-the-art model. Notably, DLM-DTI required 7.7 GB of VRAM, allowing training on conventional GPUs without the need for high-performing GPUs.

## Data Availability

The datasets are available at: https://github.com/kexinhuang12345/MolTrans/tree/master/dataset.

## Appendix A Knowledge Distillation

Recent high-performing DNN models boast millions or billions of parameters, necessitating extensive and high-performance hardware resources, such as GPU clusters and TPU pods. Knowledge distillation was proposed to develop a lightweight model while retaining robust information processing capabilities [44, 45]. The knowledge distillation process involves two models, specifically the teacher model and the student model. Conventionally, knowledge distillation begins by training the teacher model, a complex and high-capacity model, on the target task. Subsequently, the acquired knowledge from the teacher model is transferred to the student model, a more lightweight counterpart. This transfer is typically achieved by encouraging the student model to mimic the outputs [44] or internal representations [47] of the teacher model. The overarching goal is to distill the comprehensive knowledge captured by the teacher model into a more compact and computationally efficient student model.

FitNet [47] introduces the concept of “hints” to enhance the knowledge distillation approach. In addition to replicating the output of the current teacher model, hints guide the student to mimic intermediate features together. This inclusion of hints enhances the performance of knowledge distillation by enabling the learning of not only the final result but also the intermediate features. In this context, a hint can be interpreted as providing information about both the intermediate features and the final feature.

## Appendix B Drug Encoder: ChemBERTa

ChemBERTa is a Transformer-based model pre-trained using 10 million SMILES sequences [31]. Based on RoBERTa [61], a model known for its outstanding performance in natural language processing, ChemBERTa comprises 12 attention heads and 6 layers. Drug sequences, expressed in Canonical SMILES, are tokenized using a subword-level tokenizer, while a byte-pair encoder (BPE) tokenizer is employed to group frequently occurring elements together into larger chunks for more efficient processing. BPE stands as a blend of character and word-level representations, facilitating the management of extensive vocabularies in natural language corpora. Guided by the insight that less common or unfamiliar words can frequently be broken down into several recognized subwords, BPE identifies the optimal word segmentation through an iterative and greedy merging of frequently occurring character pairs [62]. ChemBERTa has a total of 767 tokens, including a class token to encapsulate the abstract meaning of the entire sequence, a start of sequence token (SOS), an end of sequence token (EOS), and a pad token to mark the start and end of the sequence.

ChemBERTa was trained using masked language modeling (MLM), where the task involves masking a portion of the entire sequence and then restoring the corresponding tokens; 15% of the total sequence was masked. The maximum processable sequence length is 512 tokens. ChemBERTa, pre-trained using MLM tasks, can then be used as an encoder for drug sequences because it has been trained on restoration tasks and has an understanding of molecule sequences. ChemBERTa can perform comparably to the commonly used extended-connectivity fingerprint (ECFP) [63] in molecule properties prediction tasks using the ChemBERTa encoder, and it was employed in this study due to its availability through the HuggingFace API, facilitating easy utilization.

## Appendix C Target Encoder: ProtBERT

ProtBERT, a component of the ProtTrans project, is a BERT model trained on an extensive dataset of amino acid sequences [35]. It underwent training using the same MLM approach as ChemBERTa, with 15% masking (Appendix B). However, owing to the intricacy of amino acid sequences, ProtBERT consists of 30 layers and 16 attention heads, resulting in a total parameter count of 4.2 million. Each element is considered one token in ProtBERT, and it comprises 30 tokens, including special tokens. Notably, it was trained to handle sequences of up to 4000 tokens, accommodating the typically extended length of amino acid sequences.

However, ProtBERT uses the Transformer’s core operation, self-attention, where the amount of computation increases as the square of the length of a given sequence. The self-attention operation is as follows:

C1 $$\begin{aligned} \text {Attention}(Q, K) = \text {softmax} \left( \frac{{QK^T}}{{\sqrt{d_k}}}\right) , \end{aligned}$$

where the query (Q) is the product of input sequence *x* and learnable parameter $W_{Q}$, and key (K) is the product of input sequence *x* and learnable parameter $W_{K}$.

Therefore, a substantial amount of memory and computational resources must be allocated to manage long sequences of amino acids. This constitutes a significant bottleneck in the practical utilization of ProtBERT. While recent proposals, such as efficient self-attention computations using linear transformers [64] and Nystrom approximation [38], aim to address this challenge, pre-training with such approaches remains expensive. As an illustration, ProtBERT underwent training utilizing 1,024 tensor processing units (TPUs), a resource allocation typically inaccessible in standard research environments. Consequently, this study emphasizes the efficient utilization of the previously published ProtBERT, prioritizing practical application over creating a new pre-training model that might reduce computational requirements.

## Code availability

The source codes are available at: https://github.com/jonghyunlee1993/DLM-DTI_hint-based-learning/tree/master.
